# Supplementary figures and images for: Effect of control measures on the pattern of COVID-19 Epidemics in Japan
Source: PeerJ. 2021 Sep 27;9:e12215. doi: 10.7717/peerj.12215 (PMC8483016; doi:10.7717/peerj.12215)

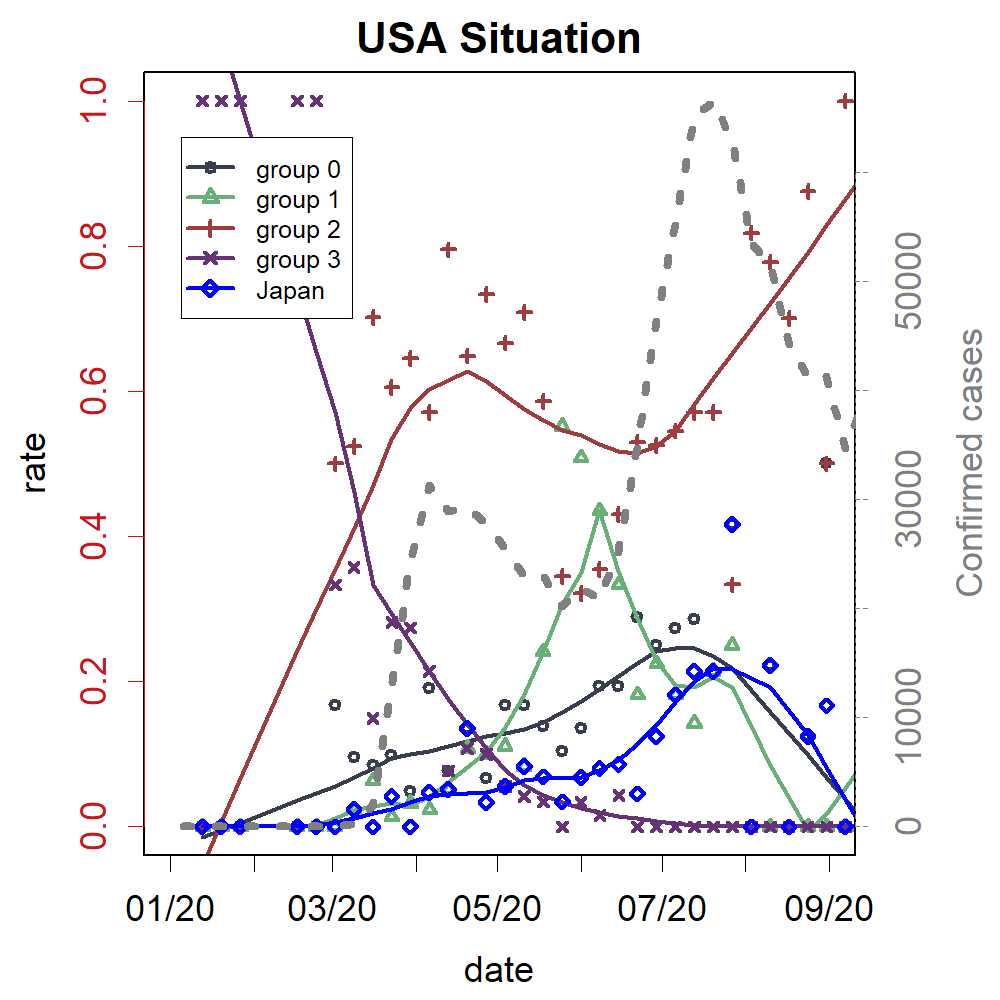

Supplement: Supplemental Information 1 — The variant that may have been exported from Japan contributed to the peak in Aug 2020 (blue). [file peerj-09-12215-s001.png]

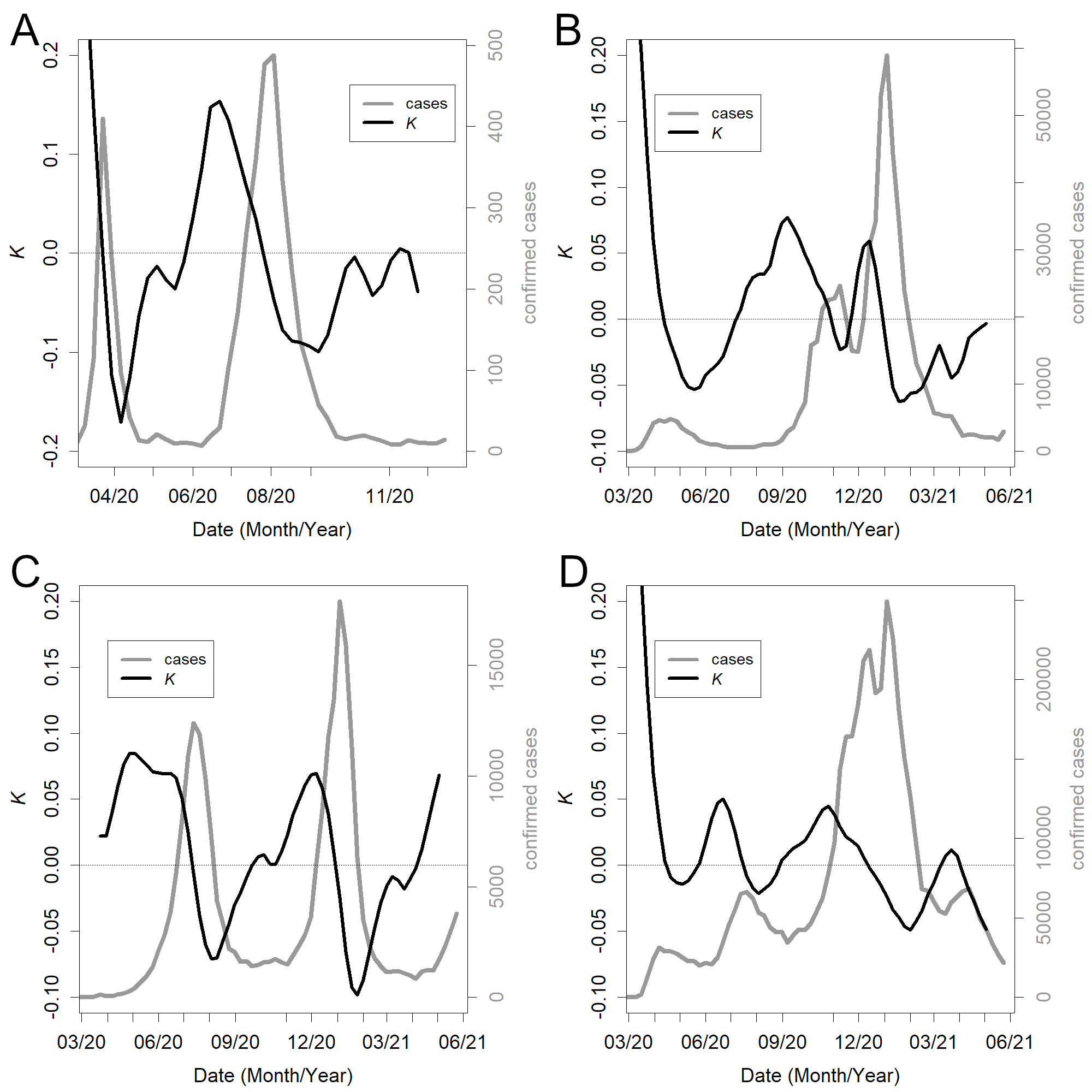

Supplement: Supplemental Information 2 — (A) Australia, (B) England, (C) South Africa, and (D) the USA. Data were obtained from World Health Organization. [file peerj-09-12215-s002.png]
